# Supplementary material for: The Removal of Pb2+ from Aqueous Solution by Using Navel Orange Peel Biochar Supported Graphene Oxide: Characteristics, Response Surface Methodology, and Mechanism
Source: Int J Environ Res Public Health. 2022 Apr 15;19(8):4790. doi: 10.3390/ijerph19084790 (PMC9032524; doi:10.3390/ijerph19084790)
Supplement: Supplementary file 1 [file ijerph-19-04790-s001.zip › ijerph-1591315-supplementary.pdf]

International Journal of
Environmental Research and Public Health

MDPI

Table S1. The adsorbent information

Adsorbent	Full name	Raw materials	Pyrolysis temperature(°C)
B300	Biochar was prepared at 300°C	Navel orange peels	300
B500	Biochar was prepared at 500°C	Navel orange peels	500
B700	Biochar was prepared at 700°C	Navel orange peels	700
GO300	Graphene oxide was prepared at 300°C	Flake graphite	300
GO500	Graphene oxide was prepared at 500°C	Flake graphite	500
GO700	Graphene oxide was prepared at 700°C	Flake graphite	700
BGO300	Biochar supported graphene oxide was prepared at 300°C	Navel orange peels and flake graphite	300
BGO500	Biochar supported graphene oxide was prepared at 500°C	Navel orange peels and flake graphite	500
BGO700	Biochar supported graphene oxide was prepared at 700°C	Navel orange peels and flake graphite	700

Table S2. Experimental design and results.

number	Experimental factors			adsorption capacity (mg/g)
	A: solution pH	B: rotating speed (r/min)	C: adsorbent dosage (g)	
1	6	150	0.005	81.35
2	4	100	0.005	75.7
3	2	100	0.0525	2.27
4	2	200	0.0525	2.77
5	4	150	0.0525	109.49
6	6	150	0.10	103.66
7	4	200	0.10	117.12
8	4	100	0.10	97.64
9	2	150	0.10	1.985
10	4	150	0.0525	110.0
11	4	150	0.0525	109.48
12	2	150	0.005	1.57
13	4	150	0.0525	108.96
14	6	100	0.0525	98.16
15	4	200	0.01	84.6
16	6	200	0.0525	100.32
17	4	150	0.0525	109.68

Solution pH, rotating speed and adsorbent dosage were used as independent variables, and Y-Pb^{2+} adsorption capacity(mg/g) was used as the dependent variable. The experimental results were analyzed by quadratic multiple regression analysis with Design-Expert 8.6 software, and the response function model of solution pH, rotating speed and adsorbent dosage on adsorption capacity was established:

$$Y=109.52+46.86A+3.88B+9.65C+0.41AB+5.47AC+2.64BC-52.63A^2-6.01B^2-9.75C^2$$

The results of significance test and variance analysis of regression model were shown in Table 4. The F-value was 107.16 and P-value < 0.0001, which indicates that the establishment of the model is extremely significant. The multivariate fitting correlation coefficient R^2 was 0.9928 > 0.8, which indicates that the model fits the data well, and the experimental error was small. In addition, the model correction coefficient R^2_{adj} was 0.9835, which indicates that the model can explain 98.35% of the change of response value, and the C.V. value was only 7.36%, which indicates that the reliability and accuracy of the experiment are high.

Table S3. Significance test and variance analysis for the quadratic regression model.

**Source of variance**	**Sum of variance**	**Degree of freedom**	**Mean square**	**F-value**	**P-value**
model	31288.05	9	3476.45	107.16	<0.0001
A	17568.94	1	17568.94	541.56	<0.0001
B	120.39	1	120.39	3.71	0.0954
C	744.75	1	744.75	22.96	0.0020
AB	0.69	1	0.69	0.021	0.8882
AC	119.86	1	119.86	3.69	0.960
BC	27.95	1	27.95	0.86	0.3842
A2	11663.72	1	11663.72	359.53	<0.0001
B2	151.99	1	151.99	4.68	0.0672
C2	400.10	1	400.10	12.33	0.0098
Residual	227.09	7	32.44	-	-
Misfit term	226.52	3	75.51	528.25	<0.0001
Pure error	0.57	4	0.14	-	-
Total value	31515.14	16	-	-	-

P<0.01 means extremely significant correlation, 0.01<p<0.05 means significant correlation, P>0.05 means not significant.
